# Chronic gastritis in China: a national multi-center survey

**DOI:** 10.1186/1471-230X-14-21

**Published:** 2014-02-07

**Authors:** Yiqi Du, Yu Bai, Pei Xie, Jingyuan Fang, Xiaozhong Wang, Xiaohua Hou, Dean Tian, Chengdang Wang, Yandi Liu, Weihong Sha, Bangmao Wang, Yanqing Li, Guoliang Zhang, Yan Li, Ruihua Shi, Jianming Xu, Youming Li, Minghe Huang, Shengxi Han, Jie Liu, Xu Ren, Pengyan Xie, Zhangliu Wang, Lihong Cui, Jianqiu Sheng, Hesheng Luo, Zhaohui Wang, Xiaoyan Zhao, Ning Dai, Yuqiang Nie, Yiyou Zou, Bing Xia, Zhining Fan, Zhitan Chen, Sanren Lin, Zhao-Shen Li

**Affiliations:** 1 Department of Gastroenterology, Changhai Hospital, Second Military Medical University, Shanghai, China; 2 Department of Gastroenterology, Renji Hospital, Shanghai Jiaotong University School of Medicine, Shanghai, China; 3 Department of Gastroenterology, Fujian Medical University Union Hospital, Fuzhou, China; 4 Department of Gastroenterology, Union Hospital, Tongji Medical College of Huazhong University of Science and Technology, Wuhan, China; 5 Department of Gastroenterology, Tongji Hospital, Tongji Medical College of Huazhong University of Science and Technology, Wuhan, China; 6 Department of Gastroenterology, First Hospital of Fujian Medical University, Fuzhou, China; 7 Department of Gastroenterology, People’s Hospital of Tianjin, Tianjin, China; 8 Department of Gastroenterology, Guangdong Provincial People’s Hospital, Guangzhou, China; 9 Department of Gastroenterology, General Hospital of Tianjin Medical University, Tianjin, China; 10 Department of Gastroenterology, Qilu Hospital of Shandong University, Jinan, China; 11 Department of Gastroenterology, First Central Hospital of Tianjin, Tianjin, China; 12 Department of Gastroenterology, Shengjing Hospital of China Medical University, Shenyang, China; 13 Department of Gastroenterology, Jiangsu Provincial People’s Hospital, Nanjing, China; 14 Department of Gastroenterology, First Hospital of Anhui Medical University, Hefei, China; 15 Department of Gastroenterology, The First Affiliated Hospital of Zhejiang University School of Medicine, Hangzhou, China; 16 Department of Gastroenterology, Shenzhen TCM Hospital, Shenzhen, China; 17 Department of Gastroenterology, Sichuan Provincial People’s Hospital, Chengdu, China; 18 Department of Gastroenterology, Huashan Hospital, Fudan University, Shanghai, China; 19 Department of Gastroenterology, Heilongjiang Provincial Hospital, Harbin, China; 20 Department of Gastroenterology, Peking University First Hospital, Beijing, China; 21 Department of Gastroenterology, Zhejiang Provincial Xinhua Hospital, Hangzhou, China; 22 Department of Gastroenterology, Navy General Hospital of PLA, Beijing, China; 23 Department of Gastroenterology, General Hospital of Beijing Military Region of PLA, Beijing, China; 24 Department of Gastroenterology, Hubei Provincial People’s Hospital, Wuhan, China; 25 Department of Gastroenterology, Dalian Central Hospital, Dalian, China; 26 Department of Gastroenterology, Xinqiao Hospital, Third Military Medical University, Chongqing, China; 27 Department of Gastroenterology, Sir Run Run Shaw Hospital, Zhejiang University School of Medicine, Hangzhou, China; 28 Department of Gastroenterology, Guangzhou First Municipal People’s Hospital, Affiliated Guangzhou Medical College, Guangzhou, China; 29 Department of Gastroenterology, Xiangya Hospital, Central South University, Changsha, China; 30 Department of Gastroenterology, Central South Hospital, Wuhan University, Wuhan, China; 31 Department of Gastroenterology, Second Affiliated Hospital, Nanjing Medical University, Nanjing, China; 32 Department of Gastroenterology, Nanjing BenQ Medical Center, Nanjing, China; 33 Department of Gastroenterology, Peking University Third Hospital, Beijing, China

**Keywords:** Chronic gastritis, Endoscopy, Epidemiology

## Abstract

**Background:**

Chronic gastritis is one of the most common findings at upper endoscopy in the general population, and chronic atrophic gastritis is epidemiologically associated with the occurrence of gastric cancer. However, the current status of diagnosis and treatment of chronic gastritis in China is unclear.

**Methods:**

A multi-center national study was performed; all patients who underwent diagnostic upper endoscopy for evaluation of gastrointestinal symptoms from 33 centers were enrolled. Data including sex, age, symptoms and endoscopic findings were prospectively recorded.

**Results:**

Totally 8892 patients were included. At endoscopy, 4389, 3760 and 1573 patients were diagnosed to have superficial gastritis, erosive gastritis, and atrophic gastritis, respectively. After pathologic examination, it is found that atrophic gastritis, intestinal metaplasia and dysplasia were prevalent, which accounted for 25.8%, 23.6% and 7.3% of this patient population. Endoscopic features were useful for predicting pathologic atrophy (PLR = 4.78), but it was not useful for predicting erosive gastritis. Mucosal-protective agents and PPI were most commonly used medications for chronic gastritis.

**Conclusions:**

The present study suggests non-atrophic gastritis is the most common endoscopic finding in Chinese patients with upper GI symptoms. Precancerous lesions, including atrophy, intestinal metaplasia and dysplasia are prevalent in Chinese patients with chronic gastritis, and endoscopic features are useful for predicting pathologic atrophy.

## Background

Chronic gastritis, a chronic inflammatory condition of gastric mucosa, is considered as one of the most common findings at endoscopy in the general population of Eastern countries [1]. In addition, *Helicobacter pylori* induced atrophic gastritis is epidemiologically associated with the occurrence of gastric cancer [2,3]. It is estimated the patients with premalignant gastric lesions carry a significant risk of gastric cancer within 10 years of follow-up, and the annual incidence of gastric cancer was 0.1% for patients with atrophic gastritis within 5 years after diagnosis [4]. Though there seems to be a worldwide decline in the overall incidence of gastric cancer, the public health burden of gastric cancer remains significant, and it is still the fourth in cancer incidence and the second leading cause of cancer-related mortality worldwide [5].

China is a country with a very high prevalence of *Helicobacter pylori* infection and gastric cancer [1], to improve the endoscopic diagnostic accuracy of chronic gastritis and standardize the diagnosis and treatment procedures of chronic gastritis, the Second Chinese National Consensus Meeting on Chronic Gastritis was held on 14th – 16th September 2006 in Shanghai, after anonymous vote according to Delphi process, the second national Chinese consensus on chronic gastritis was released [6]. However, the current status of diagnosis and treatment of chronic gastritis in China is unclear after the release of national consensus; for example, for some endoscopists, it is still a common practice to make the diagnosis of chronic atrophic gastritis just by mucosal appearance without biopsy and pathologic evidence. On the other hand, the treatment of chronic gastritis vastly varies among clinicians, and some clinicians empirically prescribe proton pump inhibitor (PPI), while some clinicians use mucosal protecting agents, occasionally natural supplements or herbal remedies are provided. Meanwhile, it remains controversial if non-ulcer patients with *Helicobacter pylori* infection shall receive eradication therapy. To address these issues, a nationwide multi-center survey was conducted to investigate the clinical, endoscopic features and treatment of chronic gastritis in Chinese patients. In addition, we aimed to determine the diagnostic accuracy of endoscopic appearance for pathologic atrophic gastritis and erosive gastritis.

## Methods

From June to December 2011, all consecutive patients aged over 18 and below 65 years old with upper GI symptoms, including epigastric pain, abdominal bloating, postprandial fullness, early satiety, belching, regurgitation, heartburn, appetite loss, nausea were screened at 33 endoscopic centers in China, and only patients who underwent first diagnostic upper endoscopy were included. Exclusion criteria were: use of PPIs, and/or non-steroidal anti-inflammatory drugs (NSAIDs), antiplatelet agents and anticoagulants, mucosal protecting agents within the previous month; history of gastrointestinal malignancy or surgery; presence of alarm features, including weight loss, hematemesis, melena or rectal bleeding, vomiting, dysphagia, anemia; pregnancy, history of drug abuse; severe cardiovascular, renal and pulmonary co-mobidities; mental illness. Patients with reflux esophagitis, peptic ulcer, polyp, and cancer at endoscopy were also excluded.

The data of the patients’ gender, age, main symptoms, endoscopic and pathologic findings were recorded. The primary and secondary symptoms were defined as we previously reported [1].

### Upper endoscopy

The present study was organized by the Chinese Society of Digestive Endoscopy, and all the 33 endoscopic centers are members of Chinese Society of Digestive Endoscopy, and all the endoscopists taking part in this investigation were well trained in a national meeting before the beginning of this study. The involved endoscopic centers had been collaborated for clinical research for a long time, and majority of the endoscopic centers in this study were also involved in other 2 Chinese national studies [7,8], therefore the agreement among these assessors was generally good.

All of the upper digestive tract were carefully examined during endoscopy. Endoscopy assessment of gastritis was based on white-light, and magnifying endoscopy or dye was not included. For controversial or non-classifiable cases, consensus was reached by discussion with one or more senior endoscopists. *Helicobacter pylori* infection was detected by pathologic examination, and site for biopsies was gastric body and gastric antrum.

All patients provided written informed consent for endoscopy before the procedure. This study was approved by the Shanghai Changhai Hospital Ethics Committee, Second Military Medical University, Shanghai, China.

### Diagnosis of chronic gastritis

The endoscopic diagnosis of chronic gastritis was consistent with the classification and grading criteria of chronic gastritis proposed by Chinese Society of Digestive Endoscopy [9]. Briefly, chronic gastritis was categorized as superficial gastritis, erosive gastritis and atrophic gastritis according to endoscopic appearance, in addition, each type of gastritis was classified into mild, moderate, and severe according to the extent of severity. In addition, dysplasia refers to phenotypically neoplastic epithelium confined to glandular structures inside the basement membrane [10].

**Figure 1:**
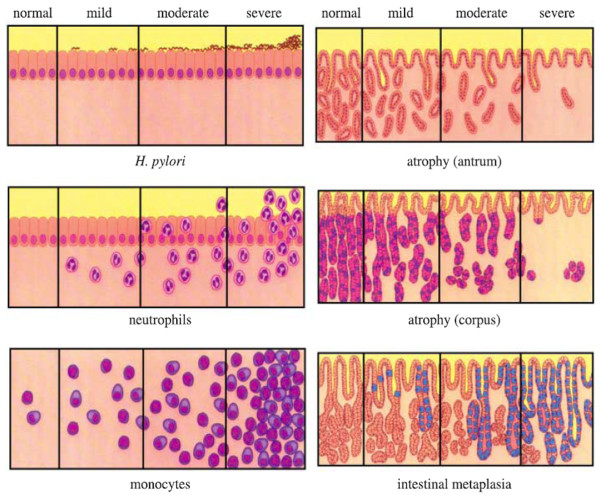
Visual analog scale for histopathology assessment.

The pathologic diagnosis of chronic gastritis was in accordance with the second national consensus on chronic gastritis [6], and pathological assessment was performed by pathologists in individual center, and these pathologists were briefed on the national consensus. They were blinded to the endoscopic diagnosis, for controversial cases, consensus was reached by discussion with one or more senior pathologists, and the agreement among these pathologists for upper GI lesions was generally good. In brief, the atrophy of gastric mucosa indicates that the gastric glands proper in the gastric mucosa become sparse. Otherwise, it would be diagnosed as non-atrophic gastritis. The criteria of histopathological grading are as follows, for grading of activity, in mild cases, the lamina propria of the mucosa is infiltrated with few neutrophils; in moderate cases, more neutrophils are seen in the mucosal layer and can also be seen in between superficial epithelial cells, pit epithelial cells and glandular epithelial cells. In severe cases a more dense infiltration of neutrophils, or abscess on pits can be seen in addition to what is seen in moderate activity. While for grading of chronic inflammation, chronic gastritis can be graded according to the density of chronic inflammatory cells. Normal: number of mononuclear cells less than 5/high power field; mild: some chronic inflammatory cells are localized in the superficial layer of the mucosa, but not more than 1/3 of the depth of the mucosa; moderate: a more dense accumulation of chronic inflammatory cells, but not exceeding 2/3 of the depth of the mucosa; severe: a dense accumulation of chronic inflammatory cells occupying the whole depth of the mucosa [6]. The histopathology assessment is summarized in Figure 1.

For patient with atrophic gastritis, if the patient was infected with *Helicobacter pylori*, then eradication therapy would be given; if the patient was not infected with *Helicobacter pylori*, then the management was similar with that of patient with non-atrophic gastritis. For patients with mild or moderate dysplasia, rigorous endoscopic surveillance program was offered, and if severe dysplasia was detected, endoscopic resection was provided. While for patients with severe dysplasia, endoscopic resection was the management of choice unless there was contraindication to endoscopic resection.

### Statistical analysis

All statistical analysis was performed by using SPSS 10.0 for Windows (Statistical Product and Service Solutions, Chicago, Illinois, USA). Categorical data was compared by chi-square test with continuity correction where appropriate. Continuous variables are expressed as mean ± standard deviation (SD), and were compared with the Student’s t-test. The diagnostic accuracy of endoscopic appearance for pathologic atrophic gastritis and erosive gastritis including sensitivity, specificity, positive predictive value (PPV), negative predictive value (NPV), positive likelihood ratio (PLR), negative likelihood ratio (NLR) and their 95% confidence interval (CI) were calculated.

## Results

From June to December 2011, totally 8897 patients from 33 centers were screened, and 5 patients refused to participate in this study after endoscopy, therefore eventually 8892 patients were included, and the cases contributed by each region are listed in Table 1. The demographic information of the patient population is shown in Table 2. The mean age of these subjects was 49.4 years old (range: 18 - 65), there were slightly more female patients than male patients (51.2% vs. 48.8%). The most common primary symptom was epigastric pain (52.9%), followed by abdominal bloating (48.7%), postprandial fullness (14.3%), and early satiety (12.7%). Among these 8892 patients, 1161 patients did not have any symptom of epigastric pain, bloating, postprandial fullness or early satiety, in the remaining patients, 53.9% had only one symptom, while the other 32.9% presented with more than one symptom (Table 3). Besides these primary symptoms, a considerable proportion of patients had complaint of secondary symptoms. Similarly, about 1/4 of these patients had more than one secondary symptom.

**Table 1:** Cases contributed by each region

**Centers**	**No.**	**%**
Shenyang	280	3.1
Dalian	500	5.6
Tianjin	964	10.8
Jinan	320	3.6
Beijing	991	11.1
Shanghai	898	10.1
Nanjing	472	5.3
Hefei	299	3.4
Hangzhou	716	8.1
Wuhan	1030	11.6
Changsha	109	1.2
Chengdu	465	5.2
Chongqing	198	2.2
Guangzhou	522	5.9
Shenzhen	295	3.3
Fuzhou	833	9.4
**Total**	**8892**	**100**

**Table 2:** Basic characteristics of the study population (n = 8892)

	**No. of patients (%)**
**Age (mean ± SD)**	49.4 ±13.2 (range 18 – 65)
**Gender**	
Male	4336 (48.8%)
Female	4556 (51.2%)
**Primary symptoms**	
Epigastric pain	4709 (52.9%)
Abdominal bloating	4328 (48.7%)
Postprandial fullness	1268 (14.3%)
Early satiety	1132 (12.7%)
**Secondary symptoms**	
Belching	2439 (27.4%)
Regurgitation	2034 (22.9%)
Heartburn	1761 (19.8%)
Appetite loss	1044 (11.7%)
Nausea	924 (10.4%)
**No. of primary symptoms**	
None	1161 (13.1%)
1	4798 (53.9%)
≥ 2	2933 (32.9%)
**No. of secondary symptoms**	
None	3393 (38.2%)
1	3480 (39.1%)
≥ 2	2019 (22.7%)

**Table 3:** Major endoscopic findings of the study population

	**No. of patients (%)**
**Superficial gastritis (n = 4389)**	**49.4%**
Mild	1845 (20.7%)
Moderate	2095 (23.6%)
Severe	449 (5.0%)
**Erosive gastritis (n = 3760)**	**42.3%**
Mild	1133 (12.7%)
Moderate	1960 (22.0%)
Severe	667 (7.5%)
**Atrophic gastritis (n = 1573)**	**17.7%**
Mild	920 (10.3%)
Moderate	576 (6.5%)
Severe	77 (0.9%)

At endoscopy, 4389, 3760 and 1573 patients were diagnosed to have superficial gastritis, erosive gastritis, and atrophic gastritis, respectively; and the majority of these lesions were mild to moderate (Table 3). After pathologic examination, it is found the presence of atrophic gastritis and intestinal metaplasia were prevalent, which accounted for 25.8% and 23.6% of this patient population, but only a small proportion of these precancerous lesions was severe. Finally, 652 patients were found to have dysplasia, which consisted of 7.3% of the whole population, and 642 (98.5%) had mild to moderate dysplasia, only 10 patients had severe dysplastic lesions (Table 4).

**Table 4:** Pathologic findings of the study population

**Atrophic gastritis (n = 2291)**	**25.8%**
Mild	1486 (16.7%)
Moderate	647 (7.3%)
Severe	158 (1.8%)
**Intestinal metaplasia (n = 2095)**	**23.6%**
Mild	1451 (16.3%)
Moderate	504 (5.7%)
Severe	140 (1.6%)
**Dysplasia (n = 652)**	**7.3%**
Mild	578 (6.5%)
Moderate	64 (0.7%)
Severe	10 (0.1%)

The diagnostic accuracy of endoscopic features for pathologic atrophic and erosive gastritis is shown in Table 5. It is noted that endoscopic appearance was not sensitive, but it was highly specific to diagnose pathologic atrophy, moreover, the PLR of endoscopic appearance for pathologic atrophy was 4.78 (95% CI: 4.33 - 5.28), suggesting patients with endoscopic atrophic appearance were more likely to have pathologic atrophy. However, endoscopic appearance was not useful for predicting neutrophils infiltration of erosive gastritis (PLR 1.18, 95% CI: 1.12 - 1.25). In addition, with a PLR of 0.86 (95% CI: 0.81 - 0.91), endoscopic appearance could not predict monocytes infiltration accurately.

**Table 5:** Diagnostic accuracy of endoscopic appearance for pathologic atrophic gastritis and erosive gastritis with pathologic results as gold standard

**Endoscopic AG (No.)**	**Pathologic AG (No.)**	**Sensitivity (95% CI)**	**Specificity (95% CI)**	**PPV (95% CI)**	**NPV (95% CI)**	**PLR (95% CI)**	**NLR (95% CI)**
1573	2291	42% (40 – 44%)	91% (91 – 92%)	69% (67 – 71%)	77% (76 – 78%)	4.78 (4.33 – 5.28)	0.64 (0.62 – 0.66)
**Endoscopic EG (No.)**	**Pathologic NI (No.)**	**Sensitivity (95% CI)**	**Specificity (95% CI)**	**PPV (95% CI)**	**NPV (95% CI)**	**PLR (95% CI)**	**NLR (95% CI)**
3760	4672	45% (43 – 46%)	62% (60 – 64%)	64% (63 – 66%)	42% (41 – 44%)	1.18 (1.12 – 1.25)	0.89 (0.86 – 0.92)
**Endoscopic EG (No.)**	**Pathologic MI (No.)**	**Sensitivity (95% CI)**	**Specificity (95% CI)**	**PPV (95% CI)**	**NPV (95% CI)**	**PLR (95% CI)**	**NLR (95% CI)**
3760	6007	41% (39 – 42%)	53% (50 – 55%)	75% (74 – 77%)	20% (19 – 21%)	0.86 (0.81 – 0.91)	1.13 (1.08 – 1.19)

AG: atrophic gastritis; EG: erosive gastritis; NI: neutrophils infiltration; MI: monocytes infiltration.

**Figure 2:**
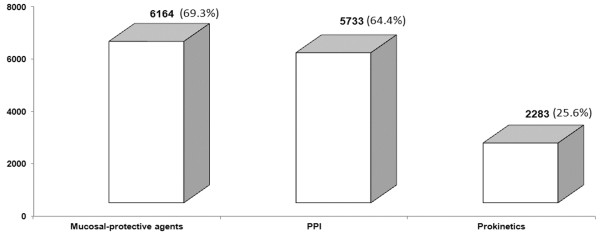
Most commonly prescribed medications for Chinese patients with chronic gastritis (n = 8892).

After endoscopic examination, the patients were prescribed with medications, and mucosal-protective agents (69.3%) and PPI (64.4%) were most commonly used (Figure 2). The Chinese traditional medicines was prescribed for 86 patients. Totally 2185 patients were diagnosed to be infected with *Helicobacter pylori* by pathologic examination; among them 1713 (78.4%) patients received *Helicobacter pylori* eradication treatment.

## Discussion

The present study suggests chronic non-atrophic gastritis is prevalent, but atrophic gastritis is also common in Chinese patients. Endoscopic atrophic appearance may predict pathologic atrophy, however, some patients with pathologic atrophy, even intestinal metaplasia, does not have typical endoscopic appearance. Further work needs to be done to identify this group of patients to detect these precancerous conditions.

This study has the following advantages, first, though chronic gastritis is very common in China, and our previous report indicated that in 1022 Shanghai residents, 63.8% had atrophic gastritis [11], but there was no large scale national study on chronic gastritis in China. Therefore, to the best of our knowledge; this study is the first and largest survey of chronic gastritis in China. Second, this national multicenter study was conducted in 33 endoscopic centers; it may represent the real-world situation of chronic gastritis in China, and the external validity of the findings of this study is credible. None the less, the present investigation has some drawbacks, the first is it is an observational study, but the large number of patients may make recompense for this limitation to some degree. Secondly, the survey was performed in 33 centers in China; so there may be clinical heterogeneity, but it can mirror the real-world practice. Finally, in this study, patients with endoscopic reflux esophagitis were excluded, however, non-erosive reflux disease (NERD) cannot be excluded.

The mean age of the patients (49.4 years old), the distribution of sex (male/female ratio: 0.95) was in line with those reported in our previous population-based study [11]. However, because of exclusion criteria, the population recruited into the study was younger and this might affect the results on atrophic gastritis due to the birth cohort effect. As regards to the symptoms, it is noted epigastric pain and abdominal bloating were very common and about half of dyspeptic patients had these two symptoms. Moreover, it is not unusual that about 40% of dyspeptic patients complained of more than just one symptom. Given the co-existence of multiple symptoms, it is suggested complex mechanisms are involved in the pathogenesis of functional dyspepsia [12].

As for the endoscopic and pathologic findings, we found the incidence of atrophic gastritis (17.7% at endoscopy and 25.8% at pathology) is much lower than that of another systematic investigation of gastrointestinal disease in Shanghai, China, in which the incidence of atrophic gastritis is 63.8% [11]. We consider the reason for this dramatic discrepancy is, first, the previous study was conducted in Shanghai only, but the present study was a multicenter national investigation. Second, the previous study applied pepsinogen (PG) I/II assay to detect atrophic gastritis, in fact, in a previous comprehensive meta-analysis looking at the validity of pepsinogen test for gastric carcinoma, dysplasia or chronic atrophic gastritis screening [13], it is found that 12 studies were aimed at diagnosing atrophic gastritis. Besides the different study designs (population-based vs. surveillance), only 8 studies addressed sensitivity and specificity. Therefore the author failed to pool the data due to the significant heterogeneity and different cut-offs. In view of this, whether atrophic gastritis can be diagnosed by pepsinogen level without upper endoscopy and pathologic examination is yet to be determined, and difference between this study and previous ones may be due to the poorly validated serological tests rather than real epidemiological difference between residents of different cities.

Though 1573 patients were endoscopically diagnosed to have atrophic gastritis, 2291 patients were found to have pathologic atrophy. We found that endoscopic appearance, such as absence of rugae, presence of visible vessels, was not sensitive to diagnose pathologic atrophy, but it was very specific for predicting pathologic atrophy, and the PLR of 4.78 indicated endoscopic atrophic appearance significantly increased the likelihood of pathologic atrophy. This finding was consistent with that of a Swedish study, which revealed that the sensitivity and specificity of endoscopic atrophic appearance for moderate to severe atrophy in gastric corpus were 48% and 87%; the corresponding values for moderate to severe atrophy in gastric antrum were 14% and 91% [14].

Besides atrophy, it is interesting to find that the prevalence of intestinal metaplasia is also high (23.6%), and it is known that intestinal metaplasia is an intermediate step in gastric carcinogenesis, and patients with intestinal metaplasia carry more than a 10-fold increased risk of developing gastric cancer [15], however currently there is no widely accepted surveillance program for intestinal metaplasia. Some experts suggest surveillance endoscopy per year may detect early gastric cancer and may improve survival [16]. Nevertheless, this surveillance program is too intensive to apply in clinical practice, in particular in countries with high prevalence of intestinal metaplasia. What is more, not all patients with intestinal metaplasia will develop cancer, so further work should be done to identify patients who are most likely to progress to cancer.

Most importantly, 7.3% of the studied population had dysplasia, though the majority patients (642 out of 652) had mild to moderate precancerous lesion, it has been established that the odds ratios (ORs) of gastric cancer increased from 17.1, for those with baseline diagnoses of superficial intestinal metaplasia, to 29.3, for those with deep intestinal metaplasia or mild dysplasia or intestinal metaplasia with glandular atrophy and neck hyperplasia, to 104.2, for those with moderate or severe dysplasia, as compared with subjects with superficial gastritis or chronic atrophic gastritis at baseline [17]. Therefore, a surveillance program is appropriate for this group of patients, who have the highest chance to progress to cancer [18].

As for the medical treatment of chronic gastritis, we found in this study, mucosal-protective agents and PPI were the most commonly prescribed medications. It is very interesting to note that there is great difference between Eastern and Western physicians in terms of the management of chronic gastritis, for instance, in Western countries, high acid output is a major etiology of upper GI disorders, so physicians in Western countries prescribe the potent anti-acid secretion drugs such as PPIs for patients [19]. However, though cumulative evidence suggested anti-acids play a major role in the treatment of gastric disorders including peptic ulcers, not all upper GI symptoms can be explained by excessive acid secretion. In particular in Asian countries, glandular atrophy is much more prevalent than that in Western societies, so low acid secretion is very common in these patients. For this reason, gastric mucosal protection is an alternative treatment for these patients. It has been reported mucosal-protective agents have different beneficial effects from PPI, for example, these agents has some biological activities for gastric mucosa, including increasing the blood flow and biosynthesis prostaglandins and the decrease of oxygen radicals [20-22]. Taken into the complex symptoms of patients with chronic gastritis into consideration, it is quite important to appropriately select medical treatment according to patient’s symptoms and pathologic findings.

Finally, 78.4% of the patients with *Helicobacter pylori* infection underwent eradication therapy. In the last 20 years, a bulk of studies has established a strong relationship between *Helicobacter pylori* infection and gastric cancer, and some experts suggest *Helicobacter pylori* screening and eradication is a cost-effective strategy for gastric cancer prevention in middle-aged adults and this strategy is especially beneficial in high-risk populations [23]. However, the available data indicate *Helicobacter pylori* eradication cannot completely prevent the occurrence of gastric cancer, and it may only be useful for patients without atrophic gastritis or intestinal metaplasia at baseline [24]. Furthermore, as the prevalence of *Helicobacter pylori* infection in China is very high (50-70%), universal *Helicobacter pylori* ‘test and treat’ strategy may not be the management of choice, and recent Asian studies also showed concern for the safety of ‘test and treat’ strategy for the management of patients with dyspepsia without alarm symptoms. For example, Sung and co-workers found 17.4% upper GI malignancies in these low-risk patients [25]. Therefore, whether patients with chronic gastritis and *Helicobacter pylori* infection shall receive eradication therapy is still an open question, and further studies are needed to clarify this issue.

## Conclusion

In summary, the findings of this present study indicate,

a) Chronic superficial gastritis and erosive gastritis are the most common endoscopic findings in Chinese patients with upper GI symptoms.

b) Precancerous lesions, including atrophy, intestinal metaplasia and dysplasia are prevalent in Chinese patients with chronic gastritis, and endoscopic atrophic appearance is useful for predicting pathologic atrophy.

c) Mucosal-protective agents and PPIs are two most commonly used medications for Chinese patients with chronic gastritis.

## Abbreviations

GI: Gastrointestinal; PPI: Proton pump inhibitor; Hp: Helicobacter pylori; SD: Standard deviation; PPV: Positive predictive value; NPV: Negative predictive value; PLR: Positive likelihood ratio; NLR: Negative likelihood ratio; CI: Confidence interval.

## Competing interests

The authors declare that they have no competing interests.

## Authors’ contributions

YD, ZL designed the study. PX, JF, XW, XH, DT, CW, YL, WS, BW, YL, GZ, YL, RS, JX, YL, MH, SH, JL, XR, PX, ZW, LC, JS, HL, ZW, XZ, ND, YN, YZ, BX, ZF, ZC, and SL participated in the design. YB conducted the data collection and carried out the statistical analysis. YB wrote the article. All authors participated in critical revision of the manuscript and have seen and approved the final version. ZL is the guarantor of this trial. All authors read and approved the final manuscript.

## Authors’ information

Yiqi Du and Yu Bai: co-first authors of this article.

## Pre-publication history

The pre-publication history for this paper can be accessed here:

http://www.biomedcentral.com/1471-230X/14/21/prepub
